# Diseases of the Lungs from Mechanical Causes, and Inquiries into the Condition of the Artisans Exposed to the Inhalation of Dust

**Published:** 1844-10

**Authors:** 


### ART. II.

*Diseases of the Lungs from Mechanical Causes, and Inquiries into the condition of the Artisans exposed to the Inhalation of Dust.* By G. CALVERT HOLLAND, M.D., Physician Extraordinary to the Sheffield General Infirmary, &c.—*London*, 1843. 8vo, pp. 100.

THE small volume bearing the above title is the fruit of—as its author announces—the labour of years; it is now presented to the public in the hope of attracting attention to the evils it describes as assailing a large proportion of the population of the town of Sheffield; and these evils may, according to the writer, be simply, readily, and effectually corrected. Here are so many reasons giving the work strong claims to attentive consideration.

The far greater number of artisans in Sheffield are grinders of cutlery and hardware; the author estimates their number at about 3000. These individuals are peculiarly subject to thoracic disease of a distinct character, and Dr. Holland has been enabled, from his position as physician to the General Infirmary of the town, to investigate its history upon an extensive scale.

Cutlery and edge-tools are all ground either upon a dry or a wet stone, or (in some instances,) upon both, the dry first, the wet afterwards,—a few articles only, such as saws and scythes, on the wet stone alone. Now, as might be anticipated, the evils of the occupation are more essentially attached to the process of dry grinding; and it is remarkable that the ill effects of its pursuit are, comparatively speaking, of recent origin and traceable to the increase of luxury in the edifices where the workmen are employed. It appears that previous to the use of steam, the grinding-wheels were invariably situated on the rivers at a distance of from two to five miles from the town, in the midst of picturesque scenery; the grinder had thus daily exercise, and abundance of pure air circulating around him; he had frequent holidays too, in consequence of the supply of water being too great or too small. Instead of all this, steam has been introduced; the "modern *grinding-wheels* are built in the town, and are several stories in height," (a local mode of expression no doubt well understood in Sheffield, but not particularly intelligible for persons living out of the grinding districts,) and no regard whatever has been bestowed upon their ventilation. Each room is occupied by eight or ten individuals belonging to different branches of the occupation, and as wheels are now erected on valuable land at a cost of from ten to twenty thousand pounds, space must be economised as far as possible, no matter how efficiently the grinder's health may be ruined by the economy. No such things as broken windows, dilapidated doors, or shattered roofs appear in these solid and well-finished structures, and hence the clouds of dust which used to escape through the slovenly, but healthful, safety-valves of former days, now accumulate in abundance round the workman's head. Such is the density of this deteriorated atmosphere that "a stranger entering the rooms at certain times, would find it difficult to breath in them."

The writer considers it extremely difficult to trace the first morbid effects of inhalation of an atmosphere surcharged with gritty and metallic particles; and the description given affords evidence of the difficulty he has felt. "The *earliest inconveniences* experienced are occasional irritation in the larynx and trachea, exciting cough and slight *expectoration, which is saliva coloured with the inhaled dust, and is sometimes quite black;* a dryness of the throat, also, generally exists." In the next paragraph it is stated that "the *first effect* of the dust is to cause an increased action in the mucous membrane of the trachea and bronchial tubes; in consequence of which, *expectoration* is excited, *presenting nothing peculiar in its character.*" We are unable to reconcile these statements. "In some cases," it is added, "when the constitution is defective in tone and vigour, showing a scrofulous or leucophlegmatic tendency, ...... purulent-like matter is observed mixed with the inhaled dust." The expectoration is said to be at first " simple saliva," yet its origin is referred to " increased action in the mucous membrane of the trachea and bronchial tubes ;" a pair of statements leading to the rather curious inference that the windpipe and its ramifications are the secreting organs of the saliva. Dr. Holland considers that the fine particles of dust do not stop short in the bronchial tubes, but pass along them to the substance of the lungs, and then give rise to structural alterations,—an opinion which is very old, but for which new reasons are adduced in the writer's pages.

Dr. Holland objects to the term grinders' asthma, by which the peculiar pulmonary disease of this class of workmen is commonly known, because " in an immense number of cases which come under observation, we do not perceive the phenomena of asthma, but of *tuberculous phthisis*." From this the reader is forced to infer that Dr. Holland regards the affection produced by the inhalation of metallic dust as tuberculous disease of the lungs.

This declaration is followed by a chapter on the " modifications in the character of phthisis, from a difference of the circumstances in which it occurs." Two or three propositions here attract our attention, less from any importance they possess in themselves, than from the very clear indications they give of the school of reasoners to which Dr. Holland belongs. " The *physiologist* would *expect* that a structural alteration in the condition of the lungs, arising from an external irritating cause, would be evolved in association with constitutional energies, very unlike what exist, when such alteration is the result of unknown vital actions." From this sentence we should judge Dr. Holland to be a person who ruminates physiology in his arm-chair, arrives at certain conclusions regarding the essence of disease, no doubt of very satisfactory character, and armed with these goes forth with a thorough-paced determination to discover at the bedside the experimental evidence of his preconceived doctrines. You may judge of a man's true character more correctly from trifles, than from the great actions of his life,—at least so hath spoken a moralist whose keenness of observation experience has taught us to deeply value. So, too, of the tone of a man's scientific mind ; chance phrases such as that we have pointed out will do more to lay it bare before us, than pages of elaborate announcement of doctrine and opinion. Now here we recognize in Dr. Holland a disciple of the *à priori* school of reasoners,—that most baneful class, which has done more for the promulgation of unsound doctrine, than it is possible to estimate. Further on Dr. Holland, speaking of " tuberculous phthisis," " contends that the *pulmonary affection springs out of a condition of the animal system prone to deterioration and decay ;* and that this is in our opinion more frequently a cause than an effect of the local malady, whether this be seated in the lungs, the kidneys, or any important gland or series of glands." We really and truly are unable to comprehend this ; how the local malady in tuberculous phthisis can be spoken of as seated in *the kidneys*, or *some or other gland*, is beyond our conception. Either Dr. Holland's notions on the subject of phthisis are curiously behind those of the day ; or here is a looseness of expression symptomatic of anything but precision of knowledge on the part of its author.

There are four chief grounds of distinction pointed out between constitutional phthisis and the " phthisis" arising from grinding cutlery.

The first is, that the latter is produced by grinding cutlery, and the former by constitutional predisposition. The next point of distinction, we are told, is one of duration. "The *average* duration of constitutional phthisis is stated," says the author, "to be almost nine months." This is an erroneous version of the fact: from the fourth to the ninth month has been found the *most common* period of death; but the *average* duration of the disease about two years. It is rather vaguely said by the author that the grinders' disease, on the contrary, will, on an average, last for years. Thirdly, the grinder, unless a consumptive diathesis exist, is, with the exception of the pulmonary affection, in good health. Fourthly, in the advanced stage of the grinders' disease there is less attenuation than in ordinary phthisis,—the breathing habitually *more* difficult, and the expectoration perhaps more copious. The pupil of the eye is seldom much dilated, nor are the teeth white. The pulse is frequently as low as eighty or eighty-five pulsations in the minute. The countenance, in this stage, exhibits great anxiety."

Chapter IV introduces us to a detailed account of "the symptoms of the disease." The "first traces of morbid action" which strike the observer are, irritation of the larynx, trachea, or bronchi, accompanied with slight and occasional cough. In persons of average vigour of constitution these symptoms excite no attention, and may continue for years without manifest aggravation; nor do they affect the general health, if they supervene in early life. If, on the contrary, they are first observed between twenty-five and thirty years of age, "the constitution is too debilitated to arrest the progress of the symptoms; consequently, these rapidly give rise to others more urgent in character, and thus one series of morbid action is quickly succeeded by another, *until at length the system breaks up*, presenting unequivocal evidence of *pulmonary phthisis.*"

In the early stage there is commonly no acceleration of pulse or fever, and the tongue rarely presents any unnatural appearance. In the next stage the cough and dyspnea increase considerably, the body is bent forwards, and "gradually sinks into less space." The appetite is good, and the digestive functions tolerably regular; the patient retains his flesh, generally speaking, to a very remarkable amount. Some quickening of pulse (*e. g.* to 75 or 80,) occurs now, but there is no hectic fever or increased heat of skin; the expectoration, chiefly mucous, may be somewhat purulent.

The chest sounds well on percussion; the respiration is natural in some few parts, puerile in others, bronchial in more.

In the third and last stage "the wretched artisan is an object painful to contemplate." The bent position of the body, and the exceedingly round shoulders constitute "one well-marked and prevailing *symptom;*" the respiration is extremely hurried, and more laborious than in ordinary phthisis; the expectoration often very copious; the mouth is not, as in the affection just named, prone to aphthous eruption, nor is the tongue smooth and polished.

The writer regards the cases in the last or advanced stage of the disease as divisible into two important classes. In the one, *cough* is the early and predominant symptom; here there is least emaciation, less tendency to diarrhea or hectic, greater oppression of breathing, and more copious expectoration; the chest is rounded anteriorly, and the sound, under percussion, particularly sonorous. The suffering and anxiety of countenance are extreme; diarrhea is seldom great.

In the other class, in which the cough is scarcely noticed by the patient, previously to laborious breathing coming on, the body wastes rapidly, the expectoration is less copious, the chest is somewhat contracted and flat, and sounds dull under percussion. "In both classes tubercles are found in the lungs, though by no means invariably in cases in which the cough was the principal distressing symptom for years."

Dr. Holland says hemoptysis occurs frequently in both classes; but is unable to state in which to the greater extent. It is said to be by no means uncommon for the grinder to bring up black, hard masses, "in appearance accretions of dust," varying in size from that of a pea to a small marble. The author knows grinders who have expectorated such masses for years, and take occasional walks (to which experience has taught them to trust,) for the purpose of facilitating their expulsion.

It is stated that the grinders are particularly subject to acute inflammatory affections—pneumonia, pleurisy, and rheumatism. The writer says that the grinder is frequently troubled with gravel; but says nothing respecting its characters.

The dulness of the sound of the chest under percussion is accompanied either with bronchial respiration, or "with a murmur as if caused by respiration performed at a distance from the surface of the lungs." The bronchial respiration the author believes to be shown, by dissection, to be the effect of enlargement of the bronchi; the second condition of respiration mentioned he "has imagined to depend on an emphysematous condition of the lungs."

The mucous membrane of the larynx, when the symptoms have existed for any length of time, becomes, in almost all cases, thickened, and is often excessively pale. The same changes take place in the mucous membrane of the bronchi and their ramifications, but to a greater extent. The membrane is often much more thickened, but usually of the same pale aspect. Dilatation of the bronchial tubes and expansion of the air-cells follow. These changes do not "prevent the subsequent production of tubercles," though these bodies "are not invariably found in the lungs." In the class of cases in which the substance of the lungs suffers first, the affection runs its course more rapidly, as has already been signified.

The differences in the morbid appearances discovered after death in the "phthisis of grinders and that occurring under ordinary circumstances" have appeared to Dr. Holland to be the following: 1. Adhesions between the lungs and pleura costalis are generally observed in the grinders' affection. We were not previously aware that such adhesions were of rare occurrence in phthisis, as Dr. Holland here leads us to suppose he considers them. 2. The next appearance which is frequently observed is great enlargement of the bronchial glands, "or, more correctly speaking, the conversion of them into a black, hard, gritty substance, varying in size from half a marble to a large hazel-nut." Dr. Holland is "not enabled to state their composition :" the idea suggests itself that he ought to have prepared himself to do so. 3. Similar substances, and "to appearance," analogous in composition, were, in several cases, detected in the substance of the lungs. And the writer states that he found them in portions of these organs which exhibited "every degree of disorganization, from the first questionable change of structure to the formation of softened tuberculous masses." 4. The lungs are frequently engorged with a black or dark fluid.

This elucidation of the "pathology" of the disease closes with a narrative of some few particulars of a case in which recovery took place. The writer comments on the fact as follows: "The point of particular interest in this case, and which, indeed, is instructive, is the *large cavity*, which was *unquestionably the cause of the unexpected recovery* of the individual thirteen years before." The notion that a "large cavity" can, *per se*, be the cause of recovery, is, to us, a very singular novelty. Many of the curious statements in the book, however, derive their originality, it is extremely probable, at least, from the looseness of the writer's style,—a looseness so remarkable that the matter is sometimes rendered quite unintelligible. Such inelegances as the following are by no means few and far between: "In the right lung an immense cavity was discovered, occupying nearly one third of *it*. An idea of *its* capacity may be formed from the fact, that the gentleman whose case *it* was, after laying *it* partly open, introduced his fist into *it*." (p. 43.) Dr. Holland is of opinion that the general dilatation of the air-tubes, which existed in this case, "perhaps contributed towards recovery, by maintaining the lungs in a condition favorable to the permeation of air." This is unquestionably a remarkable piece of therapeutical doctrine.

In considering the treatment of the affection the author maintains the division already made of the cases into two classes. In the better class, wherein the disease has not very greatly diminished the constitutional vigour of the subject, &c., the repeated application of a very few leeches, followed by blisters, with the internal use of emetics, expectorants, alteratives and tonics, is recommended by the author. In the other class of cases, "experience has not suggested any treatment which has been fraught with any large amount of benefit, beyond what is familiar to every practitioner."

Some twenty years ago the state of the grinders attracted very considerable attention, and numerous efforts were made to devise some plan by which the insalubriousness of their occupation might be lessened. It was at this time Mr. J. H. Abraham invented a magnetic mouth-piece, which was destined to attract the metallic particles evolved in the process of grinding; it of course could exercise no influence on those of the gritty kind. The invention was never much employed. Dr. Holland has laboured to invent a better, and the "trial of years" has proved the following contrivance to be "equal to the thorough correction of the evil." Here is the description of the plan:

"A wooden funnel, from ten to twelve inches square, is placed a little above the surface of the revolving stone, on the side furthest from the grinder, and this funnel terminates in a channel immediately under the surface of the floor; *or we may consider the channel simply as the continuation of the funnel*, in order to avoid any confusion in the explanation. The channel varies in length, according to the situation of the grinder, in reference to the point where it is most convenient to get quit of the dust. If we suppose that eight or ten grinders work in the same room, each has his own funnel and channel, *and they all terminate in one common channel, the capacity of which is perhaps twice or three times as great as each of the subordinate or branch channels.* The point where they terminate is always close to an external wall. At this point within the general channel, the fan is placed, somewhat in form like that used in winnowing corn, and to this is attached a strap which passes upwards, and over a pulley, so that whatever puts the pulley in motion, causes the fan also to revolve. The pulley is placed in connexion with the machinery which turns the stone, so that whenever the grinder adjusts his machinery to work, he necessarily sets the pulley and the fan in motion. The fan, acting at this point, whatever may be the length of any of the subordinate channels, causes a strong current to flow from the mouth of each funnel, which carries along with it all the gritty and metallic particles evolved, leaving the room in which the operations are pursued, free from any perceptible dust. When the whole apparatus is perfect and in excellent condition, the atmosphere is almost as healthy as that of a drawing-room."

Chapter VII, incomparably the most valuable and best of the volume, contains a full statistical account of the physical and mental condition of the artisans at Sheffield. The state of the grinders differs very considerably, according to the branch of the occupation they pursue, and for this reason the scissor-grinders, fork-grinders, needle-grinders, razor-grinders, penknife-grinders, tableknife-grinders, saw-grinders, file-grinders, and scythe-grinders, supply each of them the materials of a distinct section. We shall select some of the facts given, concerning the fork-grinders as a specimen of the whole.

Fork-grinding appears to be a peculiarly distinctive branch of the occupation. So much so that grinders belonging to other departments, frequently refuse to work in the room where it is carried on, and many sick-clubs have an especial rule against the admission of persons following it. The statistics of the branch are as follow :

| | | | |
|---|---|---|---|
| Men employed | | | 97 |
| Boys ,, | | | 100 |
| Men from ætatis | 21 to 25 | 28 | |
| ,, | 26 30 | 28 | |
| ,, | 31 35 | 8 | |
| ,, | 35 39 | 14 | |
| ,, | 40 and upwards | 19 | |
| | | — | 97 |
| Boys from ætatis | 10 to 14 | 39 | |
| ,, | 15 19 | 51 | |
| | at 20 | 10 | |
| | | — | 100 |

Of the 19 men aged above 40, there were only 3 who had reached their fiftieth year; and 10 of the 19 either commenced at this trade late in life, or passed several years in the army. Deducting these ten from the 97, there are 56 under 30 years of age; 8 from 30 to 35; 14 from 35 to 40; 9 above 40 and under 50. And in the class from 35 to 40, there are some who have not worked regularly at the trade from youth. These facts lead to the direct inference that a very considerable proportion of the whole number must die under 30 years of age. There are 56 under 30, and only 8 from 30 to 35 years of age; consequently, observes the author, the greater part of the 56 die before attaining the thirtieth year.

Between 1820 and 1825, one fourth of the total number following this branch of the occupation died. And here is a table exhibiting in a most obvious manner the enormous amount of mortality caused by this pursuit. It is entitled:

"*Comparison of deaths*, at particular ages, out of 1000 deaths of persons above 20 years of age, in England and Wales; in Sheffield generally, and of the fork-grinders particularly.

| Ages. | England and Wales. | Sheffield. | Fork grinders. |
|---|---|---|---|
| 20 to 30 | 160 | 184 | 475 |
| 30 39 | 136 | 164 | 410 |
| 40 49 | 126 | 158 | 115 |
| 50 59 | 127 | 155 | 0 |
| 20 60 | 549 | 661 | 1000 |
| All ages above 20 | 1000 | 1000 | 1090 |

"Thus," observes the author, "out of 1000 deaths of persons above 20 years of age, the proportion between 20 and 30, in England and Wales, is 160; in Sheffield 184; but amongst the fork-grinders the proportion is the appalling number 475; so that between these two periods, 3 in this trade died to 1 in the kingdom generally. Between the ages of 30 and 40, a still greater disparity presents itself. In the kingdom 136 only in the 1000 die; in Sheffield 164; but in the fork-grinding branch 410; so that between 20 and 40 years of age in this trade 885 perish out of the 1000; while in the kingdom at large, only 296. Another step in the analysis, and we perceive that between 40 and 50, in the kingdom generally 126 die; in this town 158; and in this branch 115, which completes the 1000. *They are all killed off.*"

These facts require no comment.

These unfortunate people are shown by further statements to be in a very low state of moral and intellectual condition; and to labour under a much more than average amount of disease.

This brief outline will suffice to convey some notion of the character of Dr. Holland's inquiries, and we trust tempt many to an examination of them in their complete form. Were we to form a judgment of the author from this book, we should be disposed to consider him possessed of more enthusiasm of temperament than calmness of judgment, and unquestionably more likely to reap distinction from his humanity than his pathology. But he has the best of it in the latter particular; it is impossible to close his book without conceiving a sort of personal respect for himself, and a strong desire that he may meet with becoming gratitude from the class whose condition he has so conscientiously (and successfully if there be wisdom in governments, for he has made out a clear case for the necessity of legislative interference,) laboured to ameliorate.

